# Minimally invasive coronary artery bypass grafting (MINI-CABG): Protocol for a pilot randomized controlled trial comparing minimally invasive versus conventional coronary surgery

**DOI:** 10.1371/journal.pone.0337829

**Published:** 2025-12-29

**Authors:** Luis Roberto Palma Dallan, Luiz Augusto Ferreira Lisboa, Luis Alberto Oliveira Dallan, Omar Asdrubal Vilca Mejia, Antonio Francisco Neves Filho, Torsten Doenst, Oleksandr Babliak, Gustavo Alfredo Orellana Sampedro, Fabio Biscegli Jatene

**Affiliations:** 1 Instituto do Coração, Hospital das Clínicas HCFMUSP, Faculdade de Medicina, Universidade de São Paulo, São Paulo, Sao Paulo, Brasil; 2 Cardiothoracic Surgery Department, University Hospital Jena, Friedrich Schiller University, Jena, Germany; 3 Cardiac Surgery Department, Cardiac Surgery Center, Dobrobut Medical Network, Kyiv, Ukraine,; 4 Department of Surgery, Grupo Hospitalario Kennedy, Guayaquil, Ecuador; James Cook University Hospital, UNITED KINGDOM OF GREAT BRITAIN AND NORTHERN IRELAND

## Abstract

**Introduction:**

Coronary artery bypass grafting (CABG) via sternotomy remains the standard of care for multivessel coronary disease. Minimally invasive cardiac surgery for coronary artery bypass grafting (MICS-CABG) is an evolving technique with the potential to reduce surgical trauma and promote faster recovery without compromising outcomes. This protocol describes a pilot randomized controlled trial comparing MICS-CABG and conventional CABG in multivessel patients in a high-volume tertiary center.

**Methods and analysis:**

This is a single-center, prospective, randomized controlled pilot trial. A total of 100 patients with multivessel coronary artery disease will be randomized (1:1) to undergo either conventional CABG via median sternotomy or MICS-CABG through a left anterior thoracotomy. The sample size of 100 patients (50 per group) was defined based on feasibility and statistical precision. This allows estimation of an expected 8% major adverse cardiovascular and cerebrovascular events (MACCE) rate with a 95% confidence interval half-width of ±5% in each group. These data will inform sample size calculations for a future phase III trial. The primary outcomes are safety and feasibility. Feasibility will be assessed by the successful completion of the planned minimally invasive coronary revascularization strategy. Safety will be evaluated through the occurrence of MACCE within 30 days postoperatively. Secondary outcomes include operative time, mechanical ventilation time, conversion rate to sternotomy, bleeding volume, atrial fibrillation, postoperative ICU and hospital length of stay, and patient-reported quality of life (EQ-5D-5L) 6 months postoperatively. The trial is ongoing at this moment.

**Ethics and dissemination:**

The protocol was approved by the Institutional Research Ethics Committee under que number CAAE: 54175921.8.0000.0068. Results will be disseminated through peer-reviewed publications and scientific conferences.

**Trial registration number:**

ClinicalTrials.gov NCT06794359.

## 1. Introduction

### 1.1. Background

Minimally invasive surgery has been a breakthrough in medicine, especially in more developed countries. It represents an evolution of current techniques associated with new technologic devices, that allow a safe and effective procedure associated to an aesthetic benefit.

Invasive treatment of coronary artery disease has been on a plateau lately. Percutaneous treatment that emerged as a promise to replace surgical methods, has already shown its limitations, even with the use of drug-eluting stents. Traditional CABG (coronary artery bypass grafting surgery), which has been considered the gold standard, has been criticized for its high degree of invasiveness.

Based on this precept, interest has emerged in creating a surgical approach that causes less trauma [1]. Surgical access using minimally invasive incisions are gaining space and have shown less postoperative pain, shorter hospital stays, earlier mobilization and functional recovery, in addition to reducing the costs of the procedure [2]. Recent randomized studies such as the ongoing MIST trial have further explored these patient-centered benefits, focusing on postoperative quality of life and recovery after minimally invasive coronary surgery [3].

Cardiac surgery has some challenges that are beyond other specialties. The heart is a vital organ with intrinsic chronotropism that lies inside a rigid rib cage.

Specifically in CABG, the thin caliber of the coronaries and their distribution throughout the cardiac territories creates even a greater challenge. However, new surgical techniques associated with new devices, demonstrated the viability of complete revascularization through a limited access incision.

In an attempt to minimize surgical trauma and the morbidities caused by conventional median sternotomy and the use of On Pump surgery, some alternatives have emerged with promising results.

Among those efforts, we can highlight the beginning of the coronary artery grafting surgery without the use of cardiopulmonary bypass (Off Pump CABG), followed by hybrid revascularization, minimally invasive surgery by lateral thoracotomy under direct vision, partially assisted robot surgery and fully endoscopic surgery with using robots [4].

In 1996, Calafiore published a series successfully operated on cases where CABG was performed Off Pump through the left lateral thoracotomy access. In this series, the left internal mammary artery (LIMA) was partially dissected and anastomosed to the anterior descending artery [5].

### 1.2. Historical experience in our institution

At our institution, the left mini-lateral thoracotomy and other minimally invasive access have been used in several cases to perform CABG, during its history.

In a series of 43 cases, Jatene proved that it was possible to dissect the LIMA in its full extension guided by video thoracoscope inserted through the main incision, followed by Off Pump CABG [6].

In another series of 120 cases operated on our institution and published in early 2000’s, Oliveira et al performed the treatment of single vessel coronary disease through mini access. Mortality rate was 0.8%. The conversion rate to median sternotomy was 4.2%. In an angiographic re-study foreseen in the study, the patency of the left internal thoracic artery was 98%. After 24 months of follow-up, event-free survival was 94.9% [7].

In a recent publication, 60 patients were randomized to treatment by conventional CABG surgery or by the hybrid myocardial revascularization approach. In the hybrid group, 40 patients underwent revascularization of the left anterior descending artery (LAD) using the left internal thoracic artery, using a median mini sternotomy access [8].

Currently, minimally invasive surgery though mini lateral thoracotomy is the most common technique used. Initially it was used only for the treatment of the LAD, but technology advances and surgical improvement allowed complete revascularization under direct vision.

It is possible to perform the dissection of the internal thoracic arteries in all their extension, in addition to allowing direct anastomosis of the grafts in the aorta. The method allows good visualization of the coronary arteries, especially on the left side of the heart and the posterior descending branch of the right coronary.

This current approach does not require infrastructure other than that usually offered in surgeries and is accessible to all cardiovascular surgeons. The investment to perform the surgery consists of permanent surgical material, associated with the disposable stabilizers offered by the industry [9].

In a published series of 450 minimally invasive CABG Off Pump cases, the rate of complete revascularization was 95%, with a mortality rate of 1.3%. The conversion rate to median sternotomy was 3.8% and the necessity of cardiopulmonary bypass (CPB) assist in 7.6%. The average hospital stay was 4 days [9].

Off Pump heart exposure can be challenging especially in more complex cases and during surgeon’s initial experience. In those cases, peripheral circulatory assistance for hemodynamic support, without the need for aortic cross clamping, is encouraged by experienced authors [10,11].

Off Pump graft patency in MICS CABG cases was proven to be satisfying after 6 months from surgery, with 100% LIMA to LAD patency and 92% overall graft patency [12].

Additionally, great outcomes were also published in MICS CABG using CPB with peripheral canulation, aortic cross clamping and blood cardioplegia. Complete revascularization with a mean of 3 grafts per patient and low mortality were reported in a series of 170 consecutive patients [13].

To achieve good performance, especially in initial experience, patient selection must be reasonable, choosing patients with preserved left ventricle function, feasible coronary anatomy, non-obese and without thorax deformities.

Although the 2018 ESC/EACTS guidelines on myocardial revascularization provide a IIa recommendation for minimally invasive CABG in isolated LAD lesions, it does not endorse its routine in multivessel disease once most publications are series of cases and initial centers experiences [14].

However, more recent evidence has challenged this limitation. A large systematic review encompassing approximately 7,500 patients undergoing MICS-CABG for multivessel disease demonstrated the feasibility and safety of the approach, with a high rate of complete revascularization (average of 2.8 grafts per patient) and low perioperative mortality [15].

In line with these findings, a propensity score–matched retrospective study compared minimally invasive CABG with conventional sternotomy CABG in patients with multivessel coronary disease. The authors reported comparable completeness of revascularization, graft patency, and long-term clinical outcomes between groups, with 5-year rates of MACCE and mortality remaining similar [16].

### 1.3. Rationale for the study

Minimally invasive coronary artery bypass grafting (MICS-CABG) through a left mini-thoracotomy has evolved from isolated LAD procedures to complete multivessel revascularization with excellent results in selected case series. Despite its growing adoption, the evidence supporting MICS-CABG remains largely based on single-center, non-randomized studies, limiting guideline endorsement for multivessel disease.

Recent reviews have summarized the current evidence base for minimally invasive coronary surgery, highlighting that, although MICS-CABG enables multivessel revascularization through a mini-thoracotomy without cardiopulmonary bypass, its widespread adoption remains limited because most available data come from single-center observational series. The authors emphasized that only two randomized controlled trials are currently ongoing, both led by highly experienced surgeons in high-volume centers, which may limit generalizability to the broader surgical community [17].

The MINI-CABG trial was therefore designed as a pragmatic, pilot randomized controlled study comparing MICS-CABG with conventional open CABG under contemporary surgical standards. Conducted in a South American tertiary center by surgeons undergoing structured training and progression in the minimally invasive technique, this trial aims to provide prospective, controlled data reflecting real-world conditions and to inform the design of a future large-scale phase III study.

### 1.4. MICS-CABG advantages

- Less surgical trauma
- Lower wound infection rates and mediastinitis
- Lower rates of blood transfusion
- Earlier patient mobilization in ICU
- Shorter hospital stay and faster recovery to daily activities

### 1.5. Disadvantages in MICS-CABG

- Median sternotomy conversion risk
- Cardiopulmonary pump through femoral cannulation and its peripherical inherent risks
- Possible longer CPB times and aortic cross clamp times
- When Off Pump technique is chosen, direct access to the right coronary artery becomes more challenging, although its distal branches are possible to treat.

Most of those disadvantages are related to the surgeon’s and center’s learning curves. They are usually minimized with the mastery of this new technique and a broader clinical application.

## 2. Objectives

### 2.1. Primary objective

To evaluate the feasibility and safety of performing coronary artery bypass grafting through a minimally invasive left anterior thoracotomy approach.

### 2.2. Secondary objectives

To explore differences in operative performance, postoperative recovery, complication rates, and patient-reported outcomes between minimally invasive and conventional CABG techniques.

## 3. Methods and analysis

### 3.1. Study design

This is a single-center, prospective, randomized controlled pilot trial, conducted over 30 months. A total of 100 patients will be randomized to undergo either to minimally invasive CABG through left anterior thoracotomy in the 4^th^ or 5^th^ intercostal space or conventional median sternotomy CABG. The use of CPB will be at the discreet of the surgeon.

Patients will be randomized in a 1:1 ratio using variable block sizes (blocks of 4–8), generated and managed electronically within the REDCap platform. This method ensures balance between groups while minimizing the predictability of the next allocation. The randomization module was configured by an independent researcher not involved in patient enrollment or surgery. Participants will only be randomized after meeting all eligibility criteria and signing the informed consent form.

All patients will be operated on by the same surgical team, with the standardized technique, at a high-volume tertiary cardiac surgery center.

Informed consent approved by our institutional research and ethics in committee under the number CAAE: 54175921.8.0000.0068, will be applied to all participants prior to surgery.

Post-operative follow up will be of 6 months. The postoperative evaluations will be divided into an outpatient presential evaluation around 1 month from discharge, followed by telephone contact 6 months. Follow up maybe extended up to 2 years after the surgery by telephone contact, to collect descriptive mid-term data, depending on institutional feasibility and patient availability.

**Fig 1:**
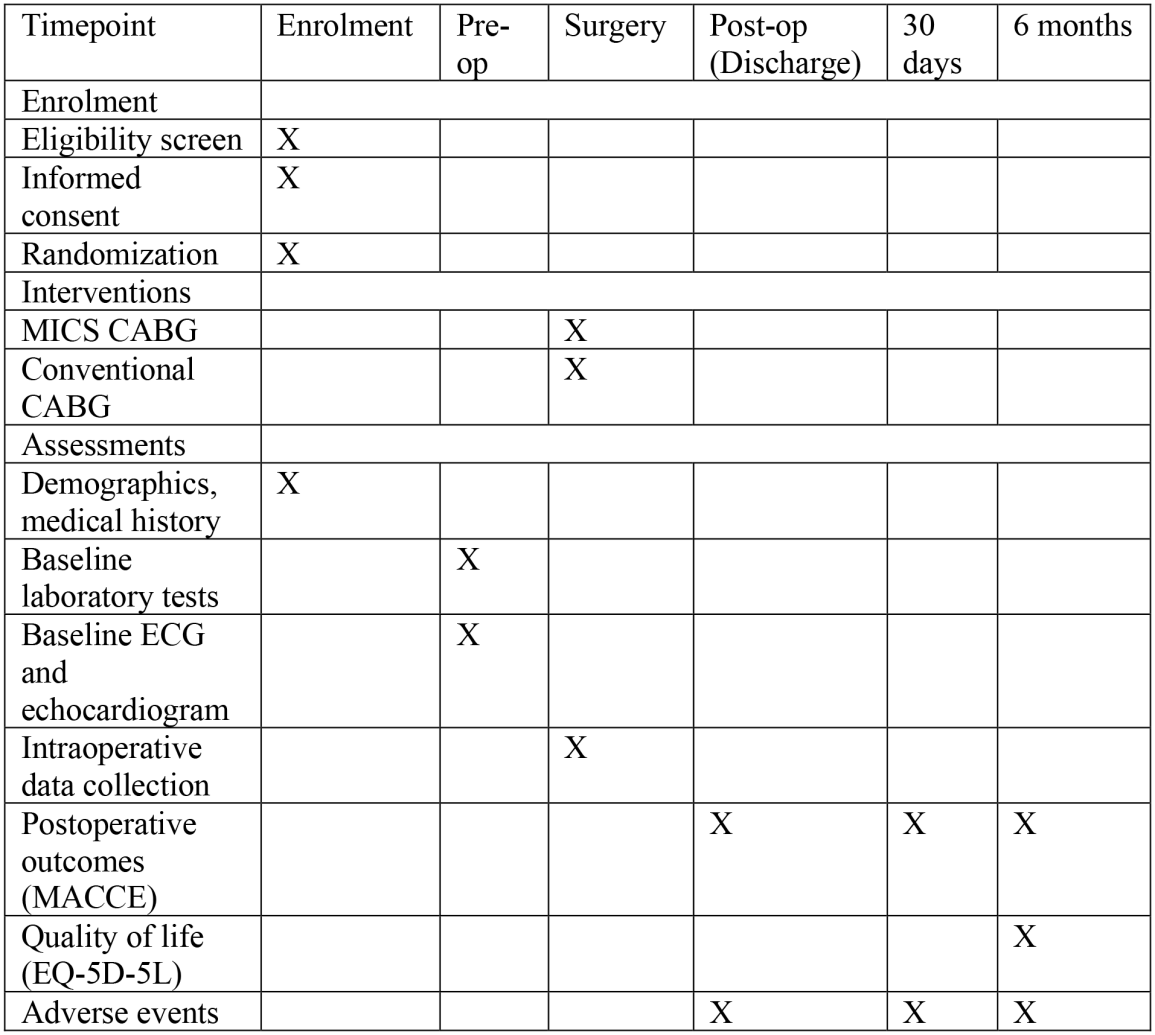
SPIRIT schedule of enrolment, interventinos, and assessments for the MINI CABG trial.

This protocol follows the SPIRIT 2013 guidelines for randomized clinical trial protocols. The SPIRIT schedule of enrolment, interventions, and assessments is shown in Fig 1.

### 3.2. Study status and timeline

The MINI-CABG trial began recruitment in October 2023. As of August 2025, a total of 60 patients have been enrolled and completed surgery, of whom 50 have completed the 6-month follow-up. Recruitment is ongoing, with an estimated total enrollment of 100 patients expected to be completed by June 2026. Data collection, including the planned follow-up period, is anticipated to be completed by January 2027. No study results have yet been generated or analyzed.

### 3.3. Ethics and dissemination

This study was approved by the Institutional Review Board of the Hospital das Clínicas, University of São Paulo (Comitê de Ética em Pesquisa do HCFMUSP – CAAE: 54175921.8.0000.0068). All participants provided written informed consent prior to enrollment. The study adheres to the principles outlined in the Declaration of Helsinki. Results will be published in peer-reviewed journals and presented at conferences.

The trial has been registered at ClinicalTrials.gov under the registration number NCT06794359.

The study received full ethics approval before patient enrollment and was registered retrospectively at ClinicalTrials.gov in October 2024, due to administrative and institutional delays during the trial initiation phase. However, we emphasize that all data have been collected prospectively, according to the predefined protocol. No outcomes were analyzed or reported prior to registration. All outcomes will be analyzed only after all patients have completed their follow-up.

This study is supported by a public grant from the São Paulo Research Foundation (FAPESP; grant 2021/11947–5). The trial also received non-financial technical collaboration from Medtronic (technical assistance and materials), without acting as a sponsor or participating in study design, data analysis, or manuscript preparation.

## 4. Participants

### 4.1. Inclusion criteria

- Patients 18-years-old or older
- Isolated myocardial revascularization procedure
- Symptomatic patients with double- or triple-vessel coronary artery disease, including proximal or mid-LAD involvement, and angiographic diameter stenosis >70% by visual analysis in all territories (or ≥50% for the left main), with suitable target vessels presenting coronary diameter ≥1.5 mm, requiring surgical myocardial revascularization; symptomatic patients with angiographic stenosis <70% by visual estimation but with invasive or non-invasive evidence of flow-limiting ischemia in all three coronary territories are also eligible for inclusion.
- Total SYNTAX score > 22 or low Syntax score unsuitable for PCI
- Clinical and anatomic eligibility for both minimally invasive and conventional CABG surgery as agreed to by surgical team.
- Silent ischemia, stable angina, unstable angina or recent MI
  - If recent MI, cardiac biomarkers must have returned to normal prior to randomization or cardiac troponin levels decreasing from a previous documented peak, consistent with the subacute phase of myocardial infarction and absence of ongoing ischemic injury.
- Ability to sign informed consent and comply with all study procedures

### 4.2. Exclusion criteria

- Reoperation
- Need for any concomitant cardiac surgery other than CABG (e.g., valve surgery, aortic repair, etc.), or intent that if the patient randomizes to surgery, any cardiac surgical procedure other than isolated CABG will be performed
- Left ventricle disfunction (EF < 40%)
- Severe Chronic Pulmonary obstructive disease defined as Severe (GOLD 3): FEV₁ 30–49% predicted
- Renal failure disease defined as estimated glomerular filtration rate (eGFR) <60 mL/min/1.73 m².
- Emergency surgery
- Obesity class II or higher (body mass index >35 kg/m²)
- Sternum anatomic defects including pectus excavatum
- Peripheral vascular disease defined as significant femoral/iliac obstructive disease precluding safe peripheral cannulation for CPB (clinical/imaging judgment)
- Ascending aorta severe calcification
- Non cardiac co-morbidities with life expectancy less than 1 year

As there is not yet an established institutional workflow specifically for MICS patients, our standard preoperative protocol does not routinely include chest CT imaging. Therefore, CT scans of the aorta and major vessels may occasionally be performed after randomization but before surgery. This occurs mainly in elective patients, due to scheduling constraints and the convenience of completing imaging during hospitalization, and in urgent cases such as patients admitted with acute coronary syndromes. If significant aortic calcification or femoral artery stenosis is identified, the patient is excluded prior to surgery and recorded as a scree failure.

This approach ensures patient safety while preserving the integrity of randomization, as no surgical intervention occurs before exclusion.

### 4.3. Eligibility assessment

Clinical and anatomic eligibility for both minimally invasive and conventional CABG is determined by the coronary surgical team. The assessment includes coronary angiographic evaluation (SYNTAX score and target vessel distribution), ventricular function, comorbidities, and overall clinical stability.

Anatomic suitability requires the presence of good distal coronary targets (diameter >1.5 mm) and, whenever predictable from coronary angiography, the avoidance of intramyocardial coronary targets, as well as the potential for achieving complete revascularization based on coronary anatomy.

Other prerequisites include the absence of significant calcification or stenosis in the ascending aorta, its major branches, and femoral arteries (to evaluate the feasibility of peripheral cannulation for cardiopulmonary bypass if required). Preoperative chest CT is reviewed to assess thoracic anatomy, lung expansion, and parenchymal conditions. Patients with sternal deformities precluding either surgical approach are excluded.

## 5. Interventions

MICS CABG vs. conventional CABG via sternotomy.

Both techniques will follow the institutional surgical standards for complete coronary revascularization. The use of on- or off-pump approach will be left to the discretion of the operating surgeon in both study arms.

Graft configuration and overall surgical strategy are similar in both groups, differing only by the access route. Arterial grafts (LIMA, RIMA, or radial) are used according to routine institutional practice, but not systematically in all cases. It depends on patient’s baseline characteristics such as age, diabetes, obesity, grade of coronary stenosis and quality of distal targets. Saphenous vein grafts and radial arteries are typically anastomosed proximally to the ascending aorta, unless intraoperative factors such as graft kinking, limited length, or an unfavorable aortic position necessitate a composite Y- or T-fashion connection to the LIMA.

### 5.1. MICS-CABG group

After anesthetic induction, patients will be intubated using a double-lumen cannula to allow single right lung ventilation. The patient will be positioned at a 30-degree lateral tilt to optimize rib spreading and lateral visualization. The surgical incision will be made in the 4th or 5th left intercostal space, approximately 8 cm in length, at the midclavicular line with medial or lateral extension if needed.

The left internal mammary artery and, if necessary, the right internal mammary artery will be dissected under direct vision using a standard electrocautery device and the Thoratrak® retractor (Medtronic), positioned with the aid of the Rultract Skyhook®. Saphenous vein and radial artery conduits will be harvested via conventional incisions.

The ascending aorta will be accessed through the left anterior mini-thoracotomy by gentle displacement toward the incision. This is achieved using cardiac tape between the aorta and pulmonary trunk or by placing a small gauze pad posteriorly to the aorta. Those maneuvers bring the aorta anteriorly within the operative field, allowing safe clamping and performance of proximal anastomoses under direct vision.

**In off-pump cases**, external suction stabilizers (Octopus Nuvo® and Starfish®, Medtronic) will be introduced through counter-incisions in the subxiphoid region and the 6th or 7th intercostal space and fixed using lateral arms attached to the operating table (Karl Storz®). Proximal anastomoses will be performed either directly on the aorta using an aortic punch (to create the opening for anastomosis) or in a Y-fashion with the LIMA. Intracoronary shunts will be used for distal anastomoses.

**In on-pump cases**, femoral artery and vein cannulation will be performed. A Chitwood aortic clamp will be introduced through the left 2nd intercostal space at the anterior axillary line. Blood cardioplegia will be administered via a long needle. After cross-clamping, tapes will be passed around the aorta, inferior vena cava, and left pulmonary veins to facilitate cardiac rotation and exposure of target vessels [18].

Intraoperative assessment of graft quality will be performed using transit time flow measurement (Medistim®). At the end of the procedure, drains will be placed through the same counter-incisions.

### 5.2. Conventional CABG group

Surgery will be performed through median sternotomy. Graft harvesting will include the left internal mammary artery, right internal mammary artery, and/or saphenous vein or radial artery, using standard techniques.

**In on-pump cases**, cardiopulmonary bypass will be established via central aortic and right atrial cannulation. Myocardial protection will be achieved using blood cardioplegia after aortic cross-clamping. Distal and proximal anastomoses will be performed under direct vision.

**In off-pump cases**, stabilization of the target coronary arteries will be performed using conventional suction stabilizers and positioning techniques. Proximal anastomoses will be performed directly on the aorta, or using composite grafts (Y-grafts), depending on anatomical feasibility.

## 6. Outcomes

### 6.1. Primary

- **Feasibility**, defined as the successful completion of the planned minimally invasive coronary revascularization strategy without conversion to sternotomy.
- **Safety**, assessed by the occurrence of major adverse cardiovascular events (MACE) within 30 days postoperatively.

### 6.2. Secondary

- **Complete revascularization rate**, defined anatomically by the treatment of all significant ischemic territories.
- **Operative metrics**, including surgery duration and intraoperative conversion rates:
  - Off-pump to on-pump conversion (in either surgical access group)
  - Mini-thoracotomy to sternotomy conversion.
- **Graft quality assessment**, using intraoperative transit time flow measurement (TTFM).
- **Postoperative recovery parameters**, including:

Duration of mechanical ventilation (hours)

- Length of ICU and hospital stay (days)
- Postoperative bleeding volume
- Blood transfusion rate.

- **Adverse events**, such as:
  - Postoperative atrial fibrillation
  - Wound infection rate
  - Stroke (type and severity)
  - Myocardial infarction (type and size, classified as procedure-related or spontaneous)
  - Cause of death (cardiac vs. non-cardiac)
- **Patient-reported outcomes**, including postoperative pain and quality of life, assessed using the EQ-5D-5L questionnaire.

Detailed descriptions of the prespecified outcomes, definitions, assessment methods, and timing are provided in S1 Table A (Study Variables and Definition).

## 7. Postoperative follow-up and data collection

Postoperative follow-up consists of an in-person visit approximately 1 month after discharge and a telephone interview at 6 months. Data collected include current medications, pain intensity on a 1–10 numerical scale, use of analgesics, rehospitalizations, and adverse events categorized as cardiovascular, neurologic, bleeding-related, vascular-access-related, infectious, or other. Mortality after discharge will also be recorded. At 6 months, patients complete the EQ-5D-5L quality-of-life questionnaire. The complete list of variables, definitions, and measurement time frames is provided in S1 Table B (Follow-up questionnaires and variables at 1 and 6 months).

## 8. Sample size and statistical analysis

This is a pilot, exploratory randomized study comparing two surgical strategies (MICS vs. conventional CABG). The primary objective is to obtain descriptive estimates of event rates (rather than on hypothesis testing), between-group differences, and variability of outcomes to guide the design of a subsequent phase III trial.

The sample size of 100 patients (50 per group) was determined by operational feasibility and expected precision, rather than hypothesis testing. Assuming a conservative 8% 30-day MACCE rate (consistent with the average reported for conventional CABG in the 2025 ESC/EACTS Guidelines) the 95% confidence interval (Wilson) has a half-width of approximately ±5% in the overall cohort and ±7–8% per arm.

This approach aligns with published pilot trials in hybrid or minimally invasive CABG, which typically include 80–120 participants and use similar precision-based rationales.

Categorical variables will be presented as frequencies and percentages, and compared using the Chi-square or Fisher’s exact test, as appropriate. Continuous variables will be expressed as mean ± standard deviation or median (interquartile range) and compared using the Student’s t-test or Wilcoxon rank-sum test. Time-to-event outcomes (e.g., MACCE-free survival) will be analyzed using the Kaplan-Meier method.

No imputation for missing data will be performed, as this is a pilot trial focused on descriptive analyses rather than formal hypothesis testing. Missing data will be reported transparently, and analyses will be conducted using available cases.

All analyses will be performed according to the intention-to-treat principle. Participants randomized to the minimally invasive CABG group who require conversion to full sternotomy will remain in the MICS CABG arm for all primary and secondary outcome analyses. Conversion events will be reported descriptively, including their timing and reasons.

The decision to use or avoid cardiopulmonary bypass (on-pump or off-pump) will be left to the operating surgeon, according to intraoperative findings.

Any conversion from an off-pump to an on-pump procedure will be documented and analyzed descriptively but will not imply a change in group allocation. If protocol deviations or conversions occur, sensitivity analyses using a per-protocol approach may be conducted to assess the robustness of the findings. The per-protocol population will include patients who received the allocated intervention as planned and completed follow-up without major protocol deviations, such as conversion to sternotomy, incorrect surgical allocation, or loss to follow-up before 30 day.

## 9. Patients and public involvement

Patients and the public were not involved in the design, conduct, reporting, or dissemination plans of this research.

**Strengths and limitations of this study**

- Minimally invasive approach is growing trend in cardiac surgery.
- This is the first randomized trial comparing MICS-CABG with conventional surgery in Latin America.
- Conducted at a high-volume center performing approximately 700 CABG procedures per year.
- Pilot design with sample size based on feasibility benchmarks from similar surgical studies, such as hybrid revascularization trials.
- The sample size is feasibility-driven and was defined based on operational and logistical considerations rather than on statistical power to detect outcome differences. This design aims to assess the study’s feasibility, recruitment process, data collection workflow, and preliminary safety signals to inform future adequately powered trials, bridging the gap between highly experienced academic centers and real-world surgical settings
- Although the center has published its experience with single vessel minimally invasive coronary artery bypass grafting (LIMA to LAD) more than 20 years ago, this is its first trial applying multiarterial minimally invasive cardiac surgery. As the surgical team progresses through a learning curve following short pre-trial training, this pilot phase is critical to assess feasibility and procedural standardization.
- The single-center design may limit generalizability to other institutions or healthcare systems.

## Supporting information

S1 Table Study variables and definitions: **Table A:** Variables and definitions; **Table B:** Follow-up questionnaires and variables at 1 and 6 months.
(DOCX)
