# Supplementary material for: Minimally invasive coronary artery bypass grafting (MINI-CABG): Protocol for a pilot randomized controlled trial comparing minimally invasive versus conventional coronary surgery
Source: PLoS One. 2025 Dec 29;20(12):e0337829. doi: 10.1371/journal.pone.0337829 (PMC12747430; doi:10.1371/journal.pone.0337829)
Supplement: S1 Table — Study variables and definitions: Table A: Variables and definitions; Table B: Follow-up questionnaires and variables at 1 and 6 months. (DOCX) [file pone.0337829.s001.docx]

# Supplementary Material 1

# Table A – Study variables and definitions

| Variable Name | Definition | Time Frame | Measurement Method |
| --- | --- | --- | --- |
| CPB or Sternotomy Conversion | Conversion defined as elective if done while hemodynamically stable; emergency if due to arrhythmias or instability. | Intraoperative | Surgical team report |
| Complete Revascularization Rate | Comparison between planned and performed grafts; incomplete if fewer grafts are done. | Intraoperative | Surgical plan vs. operative report |
| Intraoperative Graft Quality (TTFM) | Flow >15 ml/min, PI <5, Diastolic flow >50% | Intraoperative | MedStim TTFM device |
| Postoperative Bleeding | Volume in first 48h; correlated with hematocrit and transfusion | 0–48h postop | Drain output, hematocrit, transfusion records |
| Surgery Duration | Time from skin incision to skin closure | Intraoperative | Surgical log |
| Intubation Time | Time patient remains intubated postoperatively | Postoperative | Anesthesia and ICU records |
| ICU/Hospital Stay | Days from sector entry to discharge | Postoperative | Hospital records |
| Periprocedural MI | Defined by UDMI-4 and ARC-2 criteria | Within 30 days | Troponin/CK-MB, ECG, echo, angiography |
| Wound Infection | Signs of infection, purulence, lab exams within 30 days | 0–30 days postop | Clinical exam, lab results |
| Operative Mortality | All-cause mortality within 30 days postop | 0–30 days postop | Hospital and follow-up records |
| Atrial Fibrillation (Postop) | New-onset AF requiring treatment | Day 0–6mo postop (3 periods) | Clinical documentation, ECG |
| Stroke | Neurological deficit >72h confirmed by imaging | Day 0–6mo postop (3 periods) | Neurological exam, CT/MRI |
| Quality of Life (EQ-5D-5L) | Assessed at 6 months using validated questionnaire | At 6 months | Telephone survey using EQ-5D-5L |
| Follow-up | In-person evaluation at 1 month, telephone at 6 months | Up to 6 months | Outpatient visit, telephone call |

1. **Analyzed variables**

Cardiopulmonary bypass (CPB) conversion and Median sternotomy conversion rates: Conversion to CPB and/or conversion to sternotomy will be defined as elective, when the decision to made while the patient is hemodynamically stable. The conversion will be defined as an emergency if ventricular arrhythmias and/or hemodynamically instability occurs.

The surgical team will define preoperatively the number of coronary arteries to be grafted, considering **target vessels with ≥70% diameter stenosis (≥50% for left main)**and a**reference diameter ≥1.5 mm**, in accordance with guideline recommendations.

Small or ungraftable vessels are not considered in the assessment of completeness. After the surgical procedure, the number of grafts actually performed will be compared with the initial surgical plan. Incomplete revascularization will be defined when fewer grafts are performed than initially planned.

Intra operative quality evaluation of the anastomosed grafts: Intra operative time flow measurement (TTFM) allows measuring the graft flow using the ®MedStim devices. Normal flow measures are expected to be over 15ml/min, pulsatility index is expected to be lower than 5 and diastolic flow is expected to be over 50%.

Postoperative bleeding volume will be measured in the first 48 hours after surgery. It will also be correlated to pre and postoperative hematocrit values. Blood flow transfusions during surgery and 24 hours after surgery will be measured.

Surgery duration (minutes) will be timed from the skin incision until skin dressing is finished.

Length of orotracheal intubation (hours) will be timed.

Length of ICU and hospital stay (days) will be timed from patient’s entry on the sector until medical discharge is determined by intensive care and/or cardiology teams.

Periprocedural myocardial infarction (type 5 MI) will be defined according to the Fourth Universal Definition of Myocardial Infarction (UDMI-4, 2018) as an elevation of cardiac troponin values greater than 10 times the upper reference limit (URL) in patients with normal baseline levels, accompanied by at least one of the following objective indicators of myocardial ischemia: New pathological Q waves on electrocardiogram; new left bundle branch block; new regional motion abnormalities on echocardiography; angiography evidence of new occlusion of a native coronary artery or bypass graft. ^1^

To enhance methodological rigor and allow comparison with contemporary clinical trials, the periprocedural MI definition proposed by the Academic Research Consortium-2 (ARC-2) will also be adopted as a secondary criterion, which includes: Troponin elevation >70× URL, or CK-MB elevation >10× URL, in association with hemodynamic instability, need for circulatory support, or other evidence of myocardial damage.^2^

Wound infection will be defined by the presence of phlogistic signs at the incision, associated to the presence of purulent secretion and laboratorial exams, from surgery to 30-days after surgery.

Operative mortality and is defined as:

1. all deaths, regardless of cause, occurring within 30 days from the procedure (including patients transferred to other care facilities);

2. all deaths, regardless of cause, occurring after discharge from the hospital, but before the end of the 30th postoperative day (except for traumatic / external causes).

Postoperative atrial fibrillation (AF) will be defined as any new-onset episode requiring medical treatment, excluding patients with a prior or chronic history atrial fibrillation. Time Frame: AF assessment will be assessed at three time points:

- Immediate postoperative: from the day of surgery to postoperative day 7.
- Early late postoperative: from postoperative day 8 to day 30.
- Mid-term outpatient assessment: between 30 days and 6 months post-surgery.^3^

Procedure related stroke will be defined as a new neurological deficit lasting more than 72 hours, confirmed by neurological examination and brain imaging (computed tomography and/or magnetic resonance), with interpretation by a neurologist. ^4^

Stroke assessment will be performed at threetime points:

- Immediate postoperative: from the day of surgery to postoperative day 7.
- Early late postoperative: from postoperative day 8 to day 30.
- Mid-term outpatient assessment: between 30 days and 6 months post-surgery.

Postoperative quality of life will be assessed using the EuroQol EQ-5D-5L questionnaire, administered by telephone within 6 months after surgery. The EuroQol EQ-5D-5L questionnaire is a widely validated instrumental used in multicenter clinical trials, including cardiac surgery studies. Its short length, international validation, remote applicability, and utility in cost-effectiveness analyses make it ideally suited for this study.^5^

Post-operative follow up will be of 6 months. The postoperative evaluations will be divided into an outpatient presential evaluation around 1 month from discharge, followed by telephone contact 6 months after the surgery. Table 2

# Table B - Follow-up questionnaires and variables at 1 and 6 months

| **Variable** | **Question** | **Time point** | **Assessment method** |
| --- | --- | --- | --- |
| Symptoms and pain | “On a scale from 1 to 10, how would you rate your chest or incision pain in the last week?” | 1 and 6 months | Patient report: in-person / telephone |
| Analgesic use | “Have you used pain medication after hospital discharge?” (Yes/No). If yes: specify medication(s) and frequency (daily / occasional). | 1 and 6 months | Patient report: in-person / telephone |
| Medication in use | Current cardiovascular and other chronic medications (list updated). | 1 and 6 months | Review of prescription or verbal report / telephone |
| Rehospitalization | “Have you been hospitalized since your surgery?” If yes: date and cause (cardiac, neurologic, bleeding, infection, other). | 1 and 6 months | Patient report + medical records if available |
| Adverse events | Standard checklist covering cardiovascular, neurologic, bleeding, vascular, infectious, or other complications (see definitions in Table 1). | 1 and 6 months | Medical records and/or patient report |
| Mortality | Verified through hospital records or family contact. | 1 and 6 months | Hospital data or telephone confirmation |
| Quality of life | EQ-5D-5L questionnaire | 6 months | Telephone interview |

1. **Bibliographic References**

1- Thygesen K, Alpert JS, Jaffe AS, et al. Fourth Universal Definition of Myocardial Infarction (2018). Circulation. 2018;138(20):e618–e651. https://doi.org/10.1161/CIR.0000000000000617

2- Garcia-Garcia HM, McFadden EP, Farb A, Mehran R, Stone GW, Spertus J, Onuma Y, Morel MA, van Es GA, Zuckerman B, Fearon WF, Taggart D, Kappetein AP, Krucoff MW, Vranckx P, Windecker S, Cutlip D, Serruys PW; Academic Research Consortium. Standardized End Point Definitions for Coronary Intervention Trials: The Academic Research Consortium-2 Consensus Document. Circulation. 2018 Jun 12;137(24):2635-2650. doi: 10.1161/CIRCULATIONAHA.117.029289. PMID: 29891620.

3- January CT, Wann LS, Calkins H, Chen LY, Cigarroa JE, Cleveland JC Jr, et al. 2023 AHA/ACC/HRS Guideline for the Management of Patients With Atrial Fibrillation: A Report of the American College of Cardiology/American Heart Association Joint Committee on Clinical Practice Guidelines. *Circulation*. 2023;148(5):e14–e113. doi:10.1161/CIR.0000000000001150

4 - Powers WJ, Rabinstein AA, Ackerson T, Adeoye OM, Bambakidis NC, Becker K, Biller J, Brown M, Demaerschalk BM, Hoh B, Jauch EC, Kidwell CS, Leslie-Mazwi TM, Ovbiagele B, Scott PA, Sheth KN, Southerland AM, Summers DV, Tirschwell DL. Guidelines for the Early Management of Patients With Acute Ischemic Stroke: 2019 Update to the 2018 Guidelines for the Early Management of Acute Ischemic Stroke: A Guideline for Healthcare Professionals From the American Heart Association/American Stroke Association. Stroke. 2019 Dec;50(12):e344-e418. doi: 10.1161/STR.0000000000000211. Epub 2019 Oct 30. Erratum in: Stroke. 2019 Dec;50(12):e440-e441. doi: 10.1161/STR.0000000000000215. PMID: 31662037.

5- Herdman M, Gudex C, Lloyd A, Janssen MF, Kind P, Parkin D, Bonsel G, Badia X. Development and preliminary testing of the new five-level version of EQ-5D (EQ-5D-5L). Quality of Life Research. 2011 Dec;20(10):1727-36. doi:10.1007/s11136-011-9903-x.
